# Medical Treatments for Endometriosis-Associated Pelvic Pain

**DOI:** 10.1155/2014/191967

**Published:** 2014-08-07

**Authors:** Gabriella Zito, Stefania Luppi, Elena Giolo, Monica Martinelli, Irene Venturin, Giovanni Di Lorenzo, Giuseppe Ricci

**Affiliations:** ^1^Department of Medical Sciences, University of Trieste, Strada di Fiume 447, 34149 Trieste, Italy; ^2^Institute for Maternal and Child Health, IRCCS “Burlo Garofolo”, Via dell'Istria 65/1, 34137 Trieste, Italy

## Abstract

The main sequelae of endometriosis are represented by infertility and chronic pelvic pain. Chronic pelvic pain causes disability and distress with a very high economic impact. In the last decades, an impressive amount of pharmacological agents have been tested for the treatment of endometriosis-associated pelvic pain. However, only a few of these have been introduced into clinical practice. Following the results of the controlled studies available, to date, the first-line treatment for endometriosis associated pain is still represented by oral contraceptives used continuously. Progestins represent an acceptable alternative. In women with rectovaginal lesions or colorectal endometriosis, norethisterone acetate at low dosage should be preferred. GnRH analogues may be used as second-line treatment, but significant side effects should be taken into account. Nonsteroidal anti-inflammatory drugs are widely used, but there is inconclusive evidence for their efficacy in relieving endometriosis-associated pelvic pain. Other agents such as GnRH antagonist, aromatase inhibitors, immunomodulators, selective progesterone receptor modulators, and histone deacetylase inhibitors seem to be very promising, but there is not enough evidence to support their introduction into routine clinical practice. Some other agents, such as peroxisome proliferator activated receptors-*γ* ligands, antiangiogenic agents, and melatonin have been proven to be efficacious in animal studies, but they have not yet been tested in clinical studies.

## 1. Introduction

Endometriosis is a chronic disease of unknown etiology that affects approximately 10% of women in reproductive age [1]. The main sequelae of endometriosis are represented by infertility and chronic pelvic pain. Up to 40% of infertile women and one-third of women who undergo laparoscopy for chronic pelvic pain have endometriosis [1, 2]. Chronic pelvic pain causes disability and distress with a very high economic impact [3]. In the last decades several studies have been conducted in order to introduce new drugs into clinical practice for treating endometriosis-associated pelvic pain. In this paper the efficacy of older, emerging, and experimental pharmacological agents will be reviewed.

Pharmacological agents for treatment of endometriosis-associated pelvic pain are as follows.

*First-Line Treatment*. The choice should be based on patient preferences, side effects, efficacy, costs, and availability. For oral contraceptives, further benefits such as contraceptive protection, long-term safety, and control of menstrual cycle should be considered. Progestins used were medroxyprogesterone acetate, norethisterone acetate, dienogest, etonogestrel implant, and IUD-levonorgestrel (rectovaginal endometriosis).

*Second-Line Treatment*. Due to side effects, they should only be prescribed to women for whom other treatments have proven ineffective. For nonsteroidal anti-inflammatory drugs, significant side effects, including inhibition of ovulation and risk of peptic ulceration and cardiovascular disease, should be considered. For GnRH analogues significant side effects, such as bone loss and hypoestrogenic symptoms, should be considered. For danazol, severe side effects, such as thrombosis and hyperandrogenism, should be considered. For gestrinone, severe side effects, such as thrombosis and hyperandrogenism, should be considered.

*Emerging Treatments*. More clinical studies are needed, especially to assess their long-term efficacy and side effects. As for aromatase inhibitors (AIs), due to the severe side effects, they should only be prescribed to women for whom other treatments have proven ineffective. For GnRH antagonist and selective progesterone receptor modulators (SPRMs), more clinical trials are required. For histone deacetylase inhibitors, only two small pilot studies are available to date. The benefits of Selective Estrogen Receptor Modulators (SERMs) and immunomodulators have not been demonstrated to date.

*Experimental Treatments*. No clinical studies are available. For peroxisome proliferator activated receptors- (PPARs-) gamma ligands, only in vitro studies are available. For antiangiogenic agents and melatonin only animal studies are available.

## 2. Oral Contraceptives

Despite limited evidence of effectiveness, oral contraceptives are considered as first-line medical treatment for endometriosis-associated chronic pelvic pain [4, 5]; their use is based on the evidence of a clinical improvement of the disease during pregnancy [6]. They inhibit the production of gonadal estrogen via a negative feedback mechanism. Moreover, by suppressing ovarian activity, they also lead to a reduction in estrogen-induced production of prostaglandins, decreasing the inflammation associated with endometriosis. Some authors suggest the ultralow dosage [15 mcg of ethinyl estradiol (EE)], in association with 60 mcg of gestodene, for a period of 24 days, providing only 4 days off [7]. In this way, the recovery phase of estrogen synthesis (typically 7 days) is almost absent. Thus, a more stable inhibition of the production of ovarian hormones and a steady suppression of the endometrium growth are achieved [7]. In terms of side effects, lower doses of EE can lead to an increased intermenstrual bleeding, especially in the first few months of therapy, but do not affect either their contraceptive efficacy or their protective effect on endometrial and ovarian cancer and breast pathology. In a recent randomized controlled trial, it has been shown that low-dose oral contraceptive pill is more effective than placebo in controlling dysmenorrhea and in decreasing the ovarian endometrioma size [8]. The uninterrupted use of oral contraceptive pill appears to be associated with a greater pain score reduction [9]. Furthermore, the continuous administration represents a valid, safe, and economical therapeutic coverage that might be used in patients who have undergone conservative surgery for endometriosis [10, 11]. Some limitations in the use of oral contraceptives are represented by the fast recovery of the disease after the treatment interruption and the increased risk for thromboembolic events that could affect women smokers aged >35 years [12]. They can also be used together with the GnRH analogues in the “add-back therapy,” in order to balance the strong hypoestrogenism caused by the latters. In fact, they protect the bone density decrement and make women relieved of the unpleasant symptoms (above all hot flushes and vaginal discomfort) [13, 14]. However, hormone replacement treatment should be considered the first choice “add-back therapy,” since it provides lower stimulation of endometriotic tissue [5].

## 3. Progestins

Progestins have been used in the treatment of endometriosis for over 30 years. Thanks to central and peripheral mechanisms, the mitogenic action and estrogen-induced proliferation are lacking. Furthermore, the endometrium, firstly, undergoes a secretory transformation and then to a decidualization, and, finally, it becomes atrophic, thus creating a pseudopregnancy state [15, 16].

A recent Cochrane review has shown that the use of medroxyprogesterone acetate (MPA) at a dose of 100 mg/day is more effective in controlling pain if compared with placebo, but it is burdened by several side effects (menstrual irregularities, amenorrhea, weight gain, and breast tenderness) [17]. Therefore, the authors have concluded that the use of progesteron, both oral and depot form, does not seem to be more effective than the others treatments (e.g., low-dose estroprogestin or leuprolide acetate) in controlling symptoms [17].

In recent years, the use of the levonorgestrel-releasing intrauterine device (IUD-LNG) has aroused interest. Its use in the treatment of endometriosis of the rectovaginal septum provides a significant reduction in dysmenorrhea, pelvic pain, and deep dyspareunia, as well as the size of the endometriotic implants, showing levels of efficacy comparable to GnRH analogues [18, 19]. Furthermore, it appears to be effective in preventing the recurrence of endometriosis after surgical treatment [20]. Petta et al. suggested that its use would be a favourable treatment for chronic pelvic pain, because it determines a long state of hypoestrogenism, requiring only one medical intervention for its introduction every 5 years [21]. At a second-look laparoscopy, a reduction in pelvic endometriotic lesions in 60% of patients treated with LNG-IUD and in 37.5% of those treated with GnRH agonist was observed [21]. However, this difference was not significant probably due to the small sample size evaluated [21]. Clinical trials that compared the use of LNG-IUD and depot medroxyprogesterone acetate (DMPA), administered for a period of three years, showed better compliance in patients who used the IUD [22]. Moreover, bone gain was observed with LNG-IUS, whereas bone loss was reported with DMPA [22].

Recently, DMPA has been compared with an etonogestrel subcutaneous implantation (Implanon) in a pilot study [23]. During a follow-up period of 1 year, a significant decrease in pain intensity for both treatment options was observed. However, after 6 months, the average decrease in pain was 68% in the Implanon group and 53% in the DMPA group, with a side-effect profile and an overall degree of satisfaction comparable for both treatments. Therefore, Implanon might be a valid therapeutic option for pain management in women with endometriosis [23].

Norethisterone acetate (or norethindrone acetate, NETA) is a 19-nortestosterone derivative. It causes a hypoestrogenism by suppressing gonadotropins, inhibiting ovulation, and developing amenorrhea with eventual decidualization and atrophy of the endometrium [24]. Early studies showed that NETA was effective in reducing chronic pelvic pain in women with laparoscopically confirmed endometriosis [25]. Norethisterone acetate offers various advantages for the long-term treatment of endometriosis. It allows good control of uterine bleeding; it has positive effect on calcium metabolism and no negative effects on the lipoprotein metabolism at low dosages [26]. The continuous administration of NETA for the treatment of endometriosis is approved by the US Food and Drug Administration. In a randomized study on 90 women with symptomatic rectovaginal endometriosis, oral NETA 2.5 mg/day has been compared with oral EE 0.01 mg plus cyproterone acetate 3 mg/day [27]. After twelve months of continuous treatment, seven women (16%) in the EE plus cyproterone acetate arm and five (11%) in the norethindrone acetate arm had dropped out, owing to adverse effects or treatment inefficacy. In the remaining patients, dysmenorrhea, deep dyspareunia, nonmenstrual pelvic pain, and dyschezia scores were substantially reduced without major differences between the two treatment groups [27]. A prospective study on 82 women with pain symptoms caused by rectovaginal endometriosis has shown that NETA combined with letrozole was more effective in reducing pain and deep dyspareunia than NETA alone [28]. However, a higher incidence of adverse effects, without improving patients' satisfaction or influencing recurrence of pain, have been observed with the combined regimen. In a prospective study including 40 women with colorectal endometriosis who had pain and gastrointestinal symptoms, low dose of NETA (2.5 mg/day) for 12 months provided pain relief and amelioration of gastrointestinal symptoms [29].

### 3.1. Dienogest

Dienogest is a derivative of 19-nortestosterone that combines the pharmacological properties of 19-nortestosterone derivatives with those of natural progesterone derivatives [30]. Therefore, it shows a high selectivity for the progesteron receptors and a powerful progestinic effect on the endometrium [30]. Furthermore, it has beneficial antiandrogenic properties, typical of the derivatives of progesteron, which cause minimal changes in serum lipid profile and carbohydrates metabolism [31]. Currently, its oral use is approved for medical treatment of endometriosis in Europe, Japan, and Australia [31]. It has been suggested that the effectiveness of dienogest in the treatment of endometriosis depends on its ability to create a hypoestrogenic and hyperprogestinic endocrine environment, which, initially, causes the decidualization of ectopic endometrial tissue. Subsequently, for prolonged treatments, dienogest causes an atrophy of the lesions. It inhibits the estradiol level increase, through the inhibition of the growth of ovarian follicles [31]. Thus, in patients with normal ovulatory cycles, ovulation is completely suppressed. However, there are no clinical trials that have proven the contraceptive efficacy of the drug in monotherapy [32]. Regarding the optimal dose, in a European randomized controlled trial (RCT), the authors concluded that 2 mg/day represents the optimal dosage, because it is well tolerated and less encumbered by side effects, in particular abnormal uterine bleeding, and it has little influence on bone mineral density (BMD) [33]. In a recent, multicenter, randomized, double-blind study, the efficacy of dienogest 2 mg/day has been compared versus placebo. Dienogest was significantly more effective than placebo for reducing endometriosis-associated pelvic pain [34]. An open-label extension of this study for up to 53 weeks showed that long-term dienogest has a favorable efficacy and safety profile, with progressive decrease in pain and bleeding irregularities [35]. Furthermore, the decrease of pelvic pain persisted for at least 24 weeks after therapy discontinuation. These effects should be due to the multiple mechanisms of action of the drug that reduces the growth and the neoangiogenesis of the lesions and provides an anti-inflammatory activity [35]. Another RCT compared dienogest with leuprolide acetate and showed that the two drugs were equivalent in reducing pelvic pain [36]. However, dienogest was associated with a lower incidence of vasomotor symptoms and a lower impact on bone metabolism than leuprolide acetate, with a minimum change in BMD [36]. Similar results were found by using triptorelin and buserelin acetate [37, 38].

### 3.2. Danazol

Danazol is a synthetic androgen derivative of 17*α*-ethinyltestosterone, commercially introduced about 30 years ago with a specific indication for the treatment of endometriosis [39]. It carries out a multifactorial biological action inducing a hypoestrogenic-hyperandrogenic state, which is very hostile to the endometriotic tissue growth. Several studies have demonstrated the efficacy of danazol in reducing the pain associated with endometriosis [40]. However, its oral use is limited by significant side effects such as weight gain, muscle cramps, acne, seborrhoea, decreased breast size, hirsutism, and deepening of the voice, all strongly related to the androgenic action [41]. The vaginal administration, through a vaginal ring or gel or intrauterine device extended-release, has been tested in patients with deep endometriosis with encouraging results [42]. However, it should be taken into account that some data suggest that danazol may increase the risk of ovarian cancer among women with endometriosis [43].

### 3.3. Gestrinone

Gestrinone, a synthetic trienic 19-nor-steroid, acts by inhibiting the pituitary gland and, consequently, the release of gonadotrophins [44]. The resulting ovarian suppression determines atrophy of both endometrium and endometriosis lesions. It also has antiprogestinic, antiestrogenic, and androgenic action. Several studies have shown the effectiveness of gestrinone in reducing the pain associated with endometriosis [45]. However, its use is limited by the high percentage of anabolic and androgenic effects [46].

## 4. GnRH Analogues

GnRH analogues (GnRH-a) suppress estrogen ovarian production through a downregulation of GnRH receptors at pituitary level, causing a profound hypoestrogenism, and, consequently, amenorrhea and a hypoatrophic regression of the heterotopic endometrium. This effect is readily reversible after stopping GnRH-a administration. They are considered as a second-line treatment in case of failure of therapy with oral contraceptives or progestins or when they are not tolerated or contraindicated. GnRH analogues provide a reduction of symptoms in about 50% of cases [47], and their administration after surgical treatment prolongs the pain-free interval [48, 49]. The treatment for 3 months with a GnRH-a may reduce the painful symptoms for about 6 months [48]. Among the limitations of their use, there are the high rate of recurrence of pelvic pain (5 years after withdrawal of therapy is at 75%) and the side effects, such us deterioration in the lipid profile, depression, flushes, urogenital atrophy, loss of libido, and bone mass decrease [46]. The latter may be avoided by an “add-back therapy” that involves the use of hormone replacement treatment (HRT) alone or in combination with biphosphonates or other antiresorptive agents [50].

## 5. GnRH Antagonist

The use of GnRH antagonists (GnRH-anta) in the treatment of endometriosis has been recently introduced, with optimistic results [51]. They reduce estrogen levels in order to inhibit the pain symptoms but without triggering side effects consequent to estrogen deprivation. Furthermore, in contrast to GnRH-a, they do not determine the initial stimulation of the pituitary-ovarian axis with the resulting gonadotropic peak [52]. However, it is uncertain whether this specific property can actually improve the results of analogues. A recent phase 2, randomized, double-blind, placebo-controlled study has shown that a new GnRH antagonist (Elagolix) has an acceptable efficacy and safety profile [53]. More clinical trials are required before such agents should be introduced into clinical practice.

## 6. Nonsteroidal Anti-Inflammatory Drugs

Nonsteroidal anti-inflammatory drugs (NSAIDs) are the most commonly used first-line treatment for endometriosis [4, 5]. However, there is inconclusive evidence to show whether or not they are effective in relieving pain associated with endometriosis [54]. Furthermore, there is no evidence on whether any individual NSAID is more effective than another [54]. The expression of COX-2 has recently been demonstrated in ectopic endometrial cells in concentrations higher than eutopic endometrium [55]. The release of prostaglandins (PGs) in ectopic endometrial cells seems to be involved in the pathogenesis of endometriosis, and high concentrations of PGs were found in the peritoneal fluid of the affected women. Nonsteroidal anti-inflammatory drugs interfere with the function of the enzyme COX-1 and COX-2, inhibiting the production of PGs, molecules involved in the genesis of endometriosis-associated pain [55]. Specific inhibitors of COX-2, as rofecoxib, have also the property to block the growth of ectopic cells and induce apoptosis, with equivalent result to one achieved with GnRH-a [56]. Rofecoxib, at doses of 25 mg/day for 6 months, was more effective than placebo, providing an adequate and safe pain relief in cases of mild or moderate endometriosis [57]. To date, there are no sufficient clinical data to prove the NSAID drugs as effective in the treatment of endometriosis-associated pain. They are also associated with several side effects including peptic ulcer and anovulation, if taken at midcycle [55].

## 7. Aromatase Inhibitors

An overexpression of the aromatase enzyme, the main responsible factor for estrogen synthesis in ectopic endometrium, has been demonstrated in endometrial tissue [58]. Aromatase catalyzes the conversion of the steroidal precursors into estrogens, which stimulate the expression of the enzyme COX-2. The estrogens produced in the endometrial tissue through aromatase promote the growth and invasion of endometrial lesion and favour the onset of pain and prostaglandin-mediated inflammation [59]. This local production of estrogen may explain the progression of endometriosis during therapy with GnRH-a that act only at the level of the ovarian production of estrogen [60]; aromatase inhibitors (AIs), on the contrary, lead to a reduction of extraovarian estrogen concentration [61]. The estrogen plasma levels in women taking 1–5 mg of letrozole or anastrozole daily are reduced by 97–99% [62].

There are three generations of AIs. The third generation AIs, including letrozole, anastrazole, and exemestane, are triazole derivatives and have a selective, potent, and reversible action [61]. Their side effects are represented mainly by headache, stiffness or joint pains, nausea, diarrhea, and flushing. The long-term use of these drugs favours the onset of bone fractures, osteopenia, and osteoporosis [62]. However, in premenopausal women, the bone loss can be reversed by an “add-back therapy” [63, 64]. By reducing the production of extra ovarian estrogens, AIs stimulate an increased secretion of FSH from pituitary gland, promoting an increased ovarian production of estrogens and follicular recruitment. When used in premenopausal women, it is important to associate drugs that lead to a downregulation of ovarian activity, such as progestins, GnRH analogues, or oral contraceptives, in order to counteract the potential formation of follicular cysts [62–64]. The combination of conventional therapy and AIs determines the block of the production of estrogens both in ovarian and extraovarian endometriotic foci, reducing the painful symptoms. They have been used in a pilot study evaluating 12 women with rectovaginal endometriosis, who had pelvic pain resistant to conventional treatments: after 6 months of treatment with letrozole (2.5 mg/day), norethisterone acetate (2.5 mg/day), calcium citrate, and vitamin D, there has been a significant reduction in abdominal-pelvic pain and the disappearance of endometriotic lesions at second-look surgery [65]. A subsequent study, from the same group, showed that the association of letrozole with norethisterone acetate provides pelvic pain control more effectively than norethisterone acetate alone [28]. Pelvic pain, however, tends to recur after discontinuation of treatment, just as after the discontinuation of GnRH-a [66].

## 8. Selective Estrogen Receptor Modulators

Selective estrogen receptor modulators (SERMs) interact with estrogen receptors as agonists or antagonists depending on the target tissue [67]. In patients with endometriosis, the rationale for their use is related to the estrogen-antagonistic activity at endometrial level and estrogen-agonistic activity on bone and plasma lipoproteins [67]. Although studies on animals looked very promising [68, 69], currently available data in humans on SERMs do not support their clinical use. In fact, a double-blind prospective study comparing raloxifene with placebo was halted early because the raloxifene group had statistically significantly earlier pain and necessity of a second surgery [70].

## 9. Immunomodulators

According to Sampson's theory, the retrograde menstruation is one of the essential pathogenic events in the development of endometriosis. It is argued that the immune system exerts a critical role in the development of a local immune response against endometrial cells in the peritoneal cavity. Studies both in vivo and in vitro suggest that the ectopic endometrial tissue have some features that allow it to escape immunosurveillance mechanisms [71]. In particular, a significant increase of cytokines and growth factors in peritoneal fluid, some alterations of the activity of B lymphocytes, increased antibody response, and an increase of concentration and activity of peritoneal macrophages have been observed [72, 73]. Based on these concepts, the use of two different types of immunomodulators in the treatment of endometriosis has been advocated: agents capable of stimulating the cell-mediated immune response and agents capable of reducing the inflammatory response.

### 9.1. Agents Capable of Stimulating the Cell-Mediated Immune Response

Immunomodulators agents investigated in the field of endometriosis include interleukin-12 (IL-12), interferon (IFN), and two synthetic immunomodulators (the analogue of guanosine, Loxoribine, and the agonist of nicotinic cholinergic receptor, Levamisole). Interleukin-12 is a heterodimeric cytokine that acts on T and NK cells inducing the production of INF-*γ*, which in turn increases the cytotoxic activity of NK cells. A mouse model in which endometriosis was induced by the injection in the peritoneal space of syngenic endometrial fragment was developed [74]. To evaluate the ability of IL-12 to prevent the development of the disease, mice were treated with and without recombinant forms of cytokines by daily intraperitoneal injections. With an examination of the peritoneum done 3 weeks later, it was found that the overall weight and the total area of the lesions were significantly lower in mice treated with IL-12 compared to the untreated controls [74]. In order to assess the efficacy of a IL-2 in humans, Acién et al. conducted two RCT that have shown poor results both in terms of resolution of pain and reduction of endometriotic lesions [75, 76]. Ingelmo et al. proposed a murine study to test the hypothesis that the immunomodulation with recombinant human IFN-*α*-2b plays a beneficial effect on the growth of endometrial foci [77]. The result was that these cytokines, in short regimens of administration, significantly reduce endometrial implants with a long-term effect. By using a similar model of endometriosis, Keenan et al. showed that loxoribine, but not Levamisole, determines the regression of both stromal and epithelial component of the endometrial graft [78]. Unfortunately, the available data in humans are still few. Acién et al. evaluated the efficacy of IFN-*α*-2b left in the pouch of Douglas after surgery, in women with endometriosis who were undergoing conservative laparotomy [79]. They observed recurrence of the disease in 42.3% of patients treated with IFN-*α*-2b against only 15.4% of cases without interferon [79]. Thus, these results conflict with those of experimental studies. This, probably reflects the need to more fully understand the complex immunological mechanisms involved in the pathogenesis of endometriosis.

### 9.2. Agents Capable of Reducing the Inflammatory Response

Pentoxifylline is a methylxanthine with antioxidants and anti-inflammatory properties, used in the treatment of rheumatoid arthritis and inflammatory bowel disease. It works by reducing platelet aggregation through inhibition of platelet phosphodiesterase. In addition, by inhibiting the same enzyme in monocytes and macrophages, the drug suppresses the action of tumor necrosis factor- (TNF-) *α* by operating on the extracellular part of the receptor [80]. The TNF-*α* is the acute phase cytokine, involved in many processes such as apoptotic cell death, proliferation, differentiation, tumorigenesis, and viral replication. It is produced largely by macrophages and also by a number of other cell types including lymphoid cells, mast cells, endothelial cells, fibroblasts, and nerve cells. Its concentration is increased in peritoneal fluid of women with endometriosis. It has been observed that TNF-*α* can stimulate the adhesion of endometrial cells and the proliferation of ectopic and eutopic endometrial tissues in women with endometriosis [81]. Furthermore, it induces the expression of metalloproteases that favours the invasion and the angiogenesis through regulation of IL-8 expression, and it performs cytotoxic action on gametes (with a possible role in infertility) [82]. It has been demonstrated that pentoxifylline may cause suppression of endometriotic lesions by suppressing angiogenesis through vascular endothelial growth factor- (VEGF-) C and flk-1 expression [83]. Furthermore, periovulatory treatment with pentoxifylline abrogates the adverse influence of endometrial explants on fertilization in a rodent model for endometriosis [84]. Conflicting results have been obtained in human studies evaluating the effect of pentoxifylline. Some studies have concluded that there is no evidence that immunomodulation with pentoxifylline aids fertility or decreases recurrence rate of signs and symptoms in women with different stages of endometriosis [85, 86]. Other studies have demonstrated that pentoxifylline after conservative surgery for endometriosis improves VAS scores at 2 and 3 months after the procedure when compared with patients having conservative surgery only [87] and that cumulative probability of pregnancy in 6 months after laparoscopic surgery in the patients receiving pentoxifylline was higher compared with that of the patients receiving placebo [88]. A recent Cochrane review has shown that there is still not enough evidence to support the use of pentoxifylline in the management of endometriosis in terms of subfertility and relief of pain [89].

A treatment with TNF-binding protein 1 (10 mg/kg for 7 days) has been tested in a rat model [90]. A reduction of 33% and 64% in the size of endometriotic lesions, respectively, after 2 and 9 days after the end of treatment, has been observed [90]. Recent studies have reached similar conclusions using a mouse model with endometrial tissue grafts at different sites (subcutaneous tissue, peritoneum, and ovary) [91]. Treatment with anti-TNF therapy (etanercept) has been evaluated in baboon with spontaneous endometriosis [92]. Evaluating 12 baboons treated with placebo or etanercept, a significant decrease in the amount of spontaneously occurring active endometriosis was observed in animals treated with etanercept after 8 weeks of treatment [92]. It has been reported that neutralization of TNF activity with recombinant human TNFRSF1A (r-hTBP1) was as effective as GnRH antagonist in inhibiting the development of endometriosis without hypoestrogenic effects in baboons [93]. Similar results have been obtained treating baboons with a monoclonal antibody (mAb) to TNF*α* [94]. A reduction of the extention of induced peritoneal endometriosis, without interfering with the spontaneous menstrual cycle, has been observed after 25 days of treatment [94]. Only one RCT has been conducted in human [95]. This trial involving 21 participants showed no evidence of an effect of infliximab on endometriotic lesions, dysmenorrhoea, dyspareunia, or pelvic tenderness [95]. Recently, a systematic review has concluded that there is not enough evidence to support the use of anti-TNF-*α* drugs in the treatment of pelvic pain associated with endometriosis [96]. Moreover the increase of the risk of serious infections and malignancies in patients treated with long-term anti-TNF antibody therapy should be taken into account [97, 98].

## 10. Peroxisome Proliferator Activated Receptor *γ* Ligands

The peroxisome proliferator activated receptors (PPAR) represent a group of nuclear protein receptors that act as transcription factors [99]. They regulate the expression of certain genes involved in the processes of cell differentiation, development, and metabolism of carbohydrates, lipids, and proteins. PPAR*γ* ligands inhibit estrogen biosynthesis by blocking the cytochrome P450 [100]. It has, recently, been shown that PGE2 receptors EP2 and EP4 mediate actions of PPAR*γ* agonist by incorporating multiple cell signaling pathways [101]. PPAR*γ* is expressed also in endometriotic stromal cells (ESCs) [102]. Ciglitazone, pioglitazone, and rosiglitazone are thiazolidinediones, a class of compounds that show high affinity for PPAR*γ*. Experimental studies on human endometriotic cell lines and murine models have demonstrated that the activation of PPAR*γ* by ciglitazone and rosiglitazone may inhibit endometriosis proliferation [103–105] and that pioglitazone reduces the TNF-alpha-induced IL-8 production and the proliferation of ESCs [102]. The efficacy of pioglitazone has also been reported in a baboon endometriosis model [106]. The animals were treated with oral pioglitazone 7.5 mg or placebo daily for 24–42 days. The surface area and volume of endometriotic lesions were significantly lower in pioglitazone treated baboons than in the placebo group [106].

## 11. Antiangiogenic Agents

Vascular endothelial growth factor (VEGF) is the most important angiogenic factor involved in the pathogenesis of endometriosis [107]. It is a glycoprotein able to stimulate the proliferation of endothelial cells in vitro and angiogenesis in vivo. In endometriosis VEGF causes the growth of the ectopic implants. A higher level of VEGF in eutopic endometrium and red endometriotic implants of patients with endometriosis compared with healthy patients has been found; this level was correlated directly with the severity of endometriosis [108]. An increased expression of the gene encoding for VEGF in the endometrium of affected patients has also been observed [108]. Moreover, it has been shown that the peritoneal fluid from patients with endometriosis contained significantly greater amounts of VEGF than controls and that this may be critical in the pathogenesis of endometriosis [109]. In particular, only immature blood vessels (i.e., with endothelium not surrounded by pericytes) respond to VEGF. When the pericytes cover the endothelial cells, the vascular structures become mature and therefore resistant to VEGF. It has been observed that over 80% of human blood vessels present in heterotopic endometriotic lesions are pericyte-free, a percentage significantly higher than that observed in the eutopic endometrium [110]. On the basis of these considerations it has been suggested the use of antiangiogenic drugs in the treatment of endometriosis. Encouraging results have been obtained in animal models [111, 112].

## 12. Melatonin

In the peritoneal fluid of women with endometriosis an upregulation of free radicals (ROS) and a depletion of antioxidants have been observed [113]. Melatonin (N-acetyl-5-methoxytryptamine), the principal secretory product of the mammal pineal gland, is a scavenger of free radicals and it is a broad spectrum antioxidant [114]. The antioxidant, immunomodulatory, and anti-inflammatory effects of melatonin have been tested in an animal model [115]. Endometriosis was surgically induced in 25 rats; then a subgroup (*n* = 11) was treated with melatonin administered intraperitoneally, while another subgroup (*n* = 11) did not receive any treatment. Four weeks later, regression and atrophy of endometriotic lesions were noted in the melatonin-treated group only [115]. Following studies in pinealectomized mice have shown an increase of endometriotic lesions in the animals not treated with melatonin compared to those treated [116]. Furthermore, a higher concentration of molecules associated with oxidative stress such as malondialdehyde (MDA) and a statistically significant reduction of antioxidant activity were observed in pinealectomized mice [116].

## 13. Selective Progesterone Receptor Modulators (SPRMs)

Selective progesterone receptor modulators (SPRMs) represent a class of progesterone receptor ligands that display progesterone agonist, antagonist, or mixed agonist/antagonist activity on several progesterone target tissues [117]. The SPRMs were originally derived from norethindrone by the addition of a bulky substitute in the C11 position [118]. The daily administration of SPRMs induces amenorrhea through mechanisms that have not yet been fully elucidated. SPRMs inhibit the ovulation, but estradiol secretion is not affected and circulating levels of estradiol remain in the physiological range [119]. The thickening of the arterial wall might have a role in causing amenorrhea [120]. SPRM treatment induces particular endometrial changes, named PRM-associated endometrial changes (PAECs) that have not been previously observed in clinical practice [121, 122]. Endometrium of women receiving SPRM shows scanty mitosis, no atypical hyperplasia, asymmetry of stromal and epithelial growth, and prominent cystically dilated glands [121, 122]. These findings (PAECs) appear to be the result of a mixed estrogen (mitotic) and progestin (secretory) activity. Regression of endometriotic lesions with SPRM treatment has been observed in animal models [123, 124]. Few clinical studies have been so far conducted with SPRMs. An early report on 9 women showed that 50 mg of mifepristone for 6 months was effective in improving the symptoms and causing regression of endometriosis without significant side effects [125].

Asoprisnil has been tested in two randomized, placebo-controlled, dose-finding phase II studies. In the first study, women with laparoscopic diagnosis of endometriosis and having moderate or severe pain received asoprisnil (5, 10, and 25 mg) or placebo for 12 weeks [126]. All three doses of the drug resulted effectively in reduction of pain at same degree. By contrast, the effect on bleeding pattern was dose-dependent. The second study concluded that 5 mg represents the minimum effective dose for pain relief [117]. No relevant adverse events were observed during treatment or follow-up period.

A 21-substituted-19-norprogestin, Telapristone acetate (CDB-4124) [127], has also been investigated in the treatment of endometriosis [128]. Twenty-nine premenopausal women were treated with incremental oral daily doses of 12.5, 25.0, or 50.0 mg [128]. The study was aimed to evaluate only endometrial changes from a short term treatment. Endometrial biopsies, obtained after 3 or 6 months, showed histologic changes that are not seen during normal menstrual cycles. Short-term CDB-4124 treatment causes disorganized endometrial gland architecture of admixed cystic and tubular glands with nonphysiologic combinations of secretory and proliferative characteristics [128]. With increasing dose and time of administration, the endometrium becomes increasingly atrophic and the cysts more regularly prominent [128]. None of the CDB-4124-treated patients developed endometrial cancer or hyperplastic lesions during the study [128]. SPRMs are generally well tolerated. Only few cases of ovarian cysts, mostly small, asymptomatic and reversible, and dose-dependent increase of liver enzyme have been reported. Common adverse effects are headache, abdominal pain, nausea, dizziness, and metrorrhagia. In particular, headache was mainly present in the first few days of treatment, during the menstrual cycle [119].

## 14. Histone Deacetylase Inhibitors

Recently, epigenetic alterations in endometriotic cells, including the gene methylation of progesterone receptor- (PR-) B, steroidogenic factor-1 (SF-1), and estrogen receptor- (ER-) *β* have been described [129, for review]. Since demethylation agents and histone deacetylase inhibitors (HDACIs) can reactivate genes silenced by promoter hypermethylation, HDACIs have been suggested for treating endometriosis [129]. It has been shown that HDACIs trichostatin A and valproic acid can inhibit proliferation of endometrial stromal cells [129]. Moreover, it has been demonstrated that both these HDACIs reduce lesion growth and hyperalgesia in experimentally induced endometriosis in mice and rats [130, 131]. In human, acid valproic has been used for treating adenomyosis in two pilot studies [132, 133]. In the first study, after 3-month treatment, all three recruited patients reported complete disappearance of dysmenorrhea, with an average of one-third reduction in uterus size [132]. In the second pilot study 12 patients with confirmed adenomyosis, dysmenorrhea, and enlarged uterus were recruited [133]. After 3 months of valproic acid treatment, they were randomly assigned to 2 groups, one receiving no further treatment and the other the insertion of a LNG-IUS, and were followed up for an additional 3 months. At the end of the study, all patients showed complete resolution of dysmenorrhea and an average reduction in uterine size by 26%, regardless of whether LNG-IUS was used or not [133]. Thus, valproic acid seems to be a promising drug for treating adenomyosis.

## 15. Conclusion

In the last decades, an impressive amount of pharmacological agents have been tested for the treatment of endometriosis-associated pelvic pain. Some of them resulted ineffective, others proved unfit for clinical use due to significant side effects, while some others seem to be very promising but should be investigated in RCTs. Only very few have been introduced in clinical practice. Following the results of the controlled studies available, to date, the first-line treatment for endometriosis-associated pain is still represented by oral contraceptives used continuously. Progestins represent an acceptable alternative. In women with rectovaginal lesions or colorectal endometriosis, NETA at low dosage should be preferred. GnRH analogues may be used as second-line treatment, but significant side effects should be taken into account. Nonsteroidal anti-inflammatory drugs are widely used, but there is inconclusive evidence for their efficacy in relieving endometriosis-associated pelvic pain. Other agents such as GnRH antagonist, aromatase inhibitors, immunomodulators, selective progesterone receptor modulators, and histone deacetylase inhibitors seem to be very promising, but there is not enough evidence to support their introduction into routine clinical practice. Some other agents, such as peroxisome proliferator activated receptors-*γ* ligands, antiangiogenic agents, and melatonin have been proven to be efficacious in animal studies, but they have not yet been tested in clinical researches.
